# Transcriptomic Insight into Viviparous Growth in Water Lily

**DOI:** 10.1155/2022/8445484

**Published:** 2022-07-07

**Authors:** Qun Su, Hong-Yan Wang, Min Tian, Chun-Niu Li, Xian-Min Li, Zhan-Wen Huang, Zhao-Yang Bu, Jia-shi Lu

**Affiliations:** ^1^Flower Research Institute of Guangxi Academy of Agricultural Sciences, Nanning Guangxi 530007, China; ^2^Flower Research Institute of Yunnan Academy of Agricultural Sciences, Kunming Yunnan 650200, China; ^3^National Engineering Research Center for Ornamental Horticulture, Kunming Yunnan 650200, China

## Abstract

Water lily is an important ornamental flower plant which is capable of viviparous plantlet development. But no study has been reported on the molecular basis of viviparity in water lily. Hence, we performed a comparative transcriptome study between viviparous water lily *Nymphaea micrantha* and a nonviviparous species *Nymphaea colorata* at four developmental stages. The higher expression of highly conserved AUX/IAA, ARF, GH3, and SAUR gene families in *N. micrantha* compared to *N. colorata* is predicted to have a major impact on the development and evolution of viviparity in water lily. Likewise, differential regulation of hormone signaling, brassinosteroid, photosynthesis, and energy-related pathways in the two species provide clues of their involvement in viviparity phenomenon. This study revealed the complex mechanism of viviparity trait in water lily. The transcriptomic signatures identified are important basis for future breeding and research of viviparity in water lily and other plant species.

## 1. Introduction

Water lilies (*Nymphaea* spp.) are ornamental flower plants distributed worldwide from the tropics to temperate regions. They are well known and widely cultivated for environment beautification as well as water purification [1]. They have rich flower colors, long flowering periods, strong adaptability, and stress resistance and are easy to grow. As a precious flower among aquatic flowers, water lily is more popular with enthusiast breeders and botanists because of its charming appearance and unique taxonomic status. Water lilies can be classified as hardy water lilies and tropical water lilies on the basis of the typical characteristics in different ecosystems [2]. The demand for water lilies with specific traits is rapidly increasing; hence, it is required to breed new water lily varieties and hybrids with excellent ornamental characteristics. The breeders are making efforts to develop new cultivars with artificial interspecific hybridization, mutation techniques, and polyploidy approaches. Among these techniques, the hybridization is the most widely adopted method [3]. However, the existence of incongruity barriers and the development of nonviable seeds reduce the breeding efficiency and speed of multiplication [2, 3].

**Figure 1:**
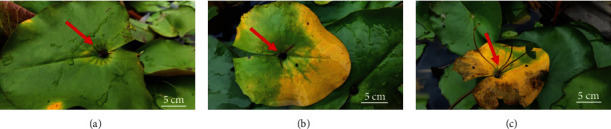
Morphological identification of viviparity in *N. micrantha.* (a–c) Different stages of the development of the viviparous structures.

The breeding through contrasting parents is a conventional tool for cultivar development in plants. In some water lily species, their leaves along with normal photosynthesis also have a reproductive function known as viviparity (Figure 1) [4]. Viviparous water lilies have the capacity to produce new plants that emerge while still attached to the parent plant known as plantlets. The slightly concave or smooth nose-like structure at the junction of the stem and leaf grows new plantlet, which can reproduce asexually in a large number of individuals in a short period of time [5]. There are a few tropical day blooming water lilies that produce plantlets from their pads. A few of the tropical night bloomers produce these plantlets from flowers [5]. The tropical day blooming variety *Nymphaea micrantha* has the ability to produce plantlets from its leaves, and it is often included in the breeding of all existing viviparous cultivars. The propagation of viviparous tropical water lilies can exponentially hasten their growth. The viviparous plantlets become mature in 20-30 days if cultivated in controlled conditions [6]. Recently, some studies on whole genome sequencing of water lilies have been reported [7, 8]. Over the past few years, application of plant molecular biotechnological tools such as pollen-tube transgenics led to a lot of achievements in improving cold stress resistance and other traits in water lilies [9]. Nonetheless, the genetic mechanism and internal metabolism of water lily leaf viviparous phenomenon are unclear.

Currently, researchers mainly focus on evolution and taxonomic status, regulation of flower color, floral fragrance, flowering period, and reproduction of water lilies [10]. With the progress in genome sequencing tools, the use of transcriptome technology may provide a new way for mining the genes related to viviparous growth in water lilies [10]. In particular, transcriptome sequencing is a useful method for identifying novel transcripts and analyzing gene expression [11]. Through RNA-seq technology, a large amount of transcript data can be generated and manipulated to evaluate the gene expression, function, and related metabolic pathways. RNA-seq or transcriptome studies have extensively been used to evaluate many plant species [12, 13] for various traits including early maturity [14] and seed dormancy [15]. The viviparity trait was investigated based on transcriptome analysis in mangrove plants [15, 16] and *Ophiorrhiza mungos* L. [4], but limited transcriptome data exists in water lilies [10].

To understand the molecular mechanism of viviparous development in water lilies, Illumina paired-end sequencing analysis of the leaf tissues from *N. micrantha* with viviparous leaves and *N. colorata* with no viviparous leaves was conducted. It is not possible to make intraspecies comparison for this trait. We reported the differentially expressed genes and their functional enrichment between both species. This comprehensive analysis of the transcriptome substantially improved the understanding of the molecular mechanisms underlying viviparous growth in plant.

## 2. Materials and Methods

### 2.1. Plant Materials, Growth Conditions, Morphological Identification, and RNA Extraction

**Figure 2:**
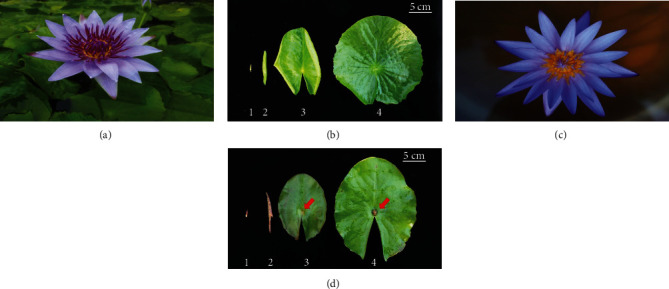
Flowers of nonviviparous *N. colorata* (a and b) and viviparous *N. micrantha* (c and d). The red arrows indicate the viviparous structure, and numeric 1 to 4 in (b) and (d) indicated the four developmental stages.

The plants of two *Nymphaea* species *N. micrantha* (denoted with “X” in this study) and *N. colorata* (denoted with “L” in this study) (Figures 2(a) and 2(b)) were grown in a planting container of 100 cm diameter and 60 cm depth, with 30 cm soil thickness with standard natural growth conditions at Water Lily Resource Garden of the Floriculture Institute of Guangxi Academy of Agricultural Sciences, Guangxi, China, during the growth season of 2019. Six disease-free plants with robust growth for each genotype were selected for the transcriptome study. The morphological identification was performed when the leaves were fully unfolded and a clear viviparous mark in center could be observed. The leaf sampling was performed at four growth stages as (1) life size 1-2 cm long, (2) leaf size 4-6 cm, (3) when leaf tip connected to water surface, and (4) fully developed leaves. The fresh leaf samples with the leaf nose parts were collected at four developmental stages (Figures 2(c) and 2(d)). The leaves from six plants of the same genotype as technical replicates were mixed for each sample to make a homogenized sample, and a total of three biological repeats were obtained. The harvested leaves were immediately frozen in liquid nitrogen and stored at -80°C.

### 2.2. RNA Extraction and Preparation of Library

The total RNA was extracted by CTAB method [17] from leaf tissues of each sample using the three biological repeats. The extracted RNA was further evaluated for concentration (by NanoDrop 2000 microspectrophotometer), purity (by Agilent 2100 Bioanalyzer), and integrity in agarose gel. The step-by-step process as total RNA sample detection, mRNA enrichment with Oligo (dT) beads, fragmentation with fragmentation buffer, synthesis of double-stranded cDNA, purification and end repair, splice selection and PCR amplification library quality detection, and the computer-based sequencing was performed at Entrusted Jiugui Biotechnology Company Limited, Shanghai, China, and finally, the paired-end reads were generated.

### 2.3. Transcriptome Sequencing, Cleaning, and Assembly

The original image data files were obtained by high-throughput sequencing (Illumina HiSeq 4000) and were transformed into raw reads by base calling analysis. As per machine's sequencing strategy, 150 bp average read length was maintained. Raw data were processed using NGS QC Toolkit [18]. The raw reads with joint sequences, and/or less than 5 mass value, more than or equal to 50% proportion rate, more than or equal to 5% N-base (the base with undetermined information), containing Poly-A were filtered out to get the cleaned reads. Because the reference genome of *N. micrantha* is not yet available, we decided to a de novo assembly of the transcriptomes of the two species in this study. The Trinity v 2.6.6 program [19] was used for transcriptome assembly and to get the unigenes. The accuracy and effectiveness of the assembly results were ensured by estimating the N50 and exN50.

### 2.4. Expression Evaluation and Identification of Differentially Expressed Genes

The number of reads count on each gene was obtained from each sample, and the gene expression level was estimated by the fragments per kilobase of transcript per million mapped reads (FPKM) method. FPKM value of each gene was calculated using cufflinks [20], and the read counts of each gene were obtained by htseq-count [21]. Differentially expressed genes (DEGs) were identified using the DESeq (with replicates) [22]. *P* value < 0.05 and log2 fold change > 1 for upregulated and fold change < −1 for downregulated DEGs were set as the threshold for significant differential expression. Principal component analysis of DEGs was performed to explore the gene expression pattern.

### 2.5. Functional Annotation and Enrichment Analysis

The extracted unigenes were manipulated by Transcoder software v 4.1.0 to predict and translate the reading frames. Gene Ontology (GO) [23] enrichment and Kyoto Encyclopedia of Genes and Genomes (KEGG) pathway [24] enrichment analysis of DEGs were, respectively, performed using R based on the hypergeometric distribution. Blast2go [25] and Kaas software [26] (https://www.genome.jp/tools/kaas/) tools were used for GO and KEGG annotation, respectively, and Phyper function in R software was used for enrichment analysis. The gene expression was determined by comparing the sequenced reads with the unigene library in Bowtie [27].

### 2.6. Expression Validation by Quantitative Real-Time PCR

Ten DEGs were further evaluated by qRT-PCR. The quantitative real-time PCR was performed using SYBR green mixture on an ABI 7500 real-time PCR detection system following the descriptions of Komivi et al. [28]. The *Ubiquitin* gene was used as internal control for normalization. The statistical approach developed by Livak and Schmittgen [29] was employed for statistical analysis. The expression data was further evaluated by Student's *t*-test for significance estimation. All primer sequences are listed in Additional Table S4.

## 3. Results

### 3.1. Morphological Indication of Viviparity

The leaf tissues were evaluated at four developmental stages of the viviparous species *N. micrantha* denoted as X and nonviviparous species *N. colorata* denoted as L (Figure 2). The four developmental stages can be defined as follows: stage (1) when the leaves are submerged and completely rolled with 1-2 cm length, stage (2) when the leaves are submerged and completely rolled with 4-6 cm length, stage (3) when the leaves are half rolled with the tip in contact with water surface, and stage (4) when the leaves are fully unfolded with obvious viviparous traces/mark in the center of X-species while absent in L-species (Figures 2(b) and 2(d)). The viviparous structure was observable from the 2^nd^ growth stage while obvious at the 3^rd^ and 4^th^ stages.

### 3.2. Transcriptome Assembly for Water Lily Species

For a comprehensive insight into the genes related to development of vivipary trait in water lilies, leaf samples at four developmental stages of both X- and L-species were collected (Figure 2). The cDNA libraries were constructed from three biological repeats. The high-throughput sequencing (Illumina HiSeq 4000 platform) data was generated and then transformed into the raw data by base calling analysis. A maximum 58.69 and 61.75 million raw reads were extracted for X and L, respectively (Table 1). After cleaning the reads, maximum 8484.73 and 8868.31 million bases with 49.5% and 49% GC contents and 94.98% and 94.81% *Q* > 30 were retained for X and L, respectively (Table 1). The assembly of clean reads provided 114,762 unigenes with an average length of 866.87 bp (Table 2). All the screened unigenes were larger than 300 bp size, while 27.59% unigenes (31,663) displayed extralong size (>1,000 bp) (Table 2). The high expression quality (ExN50) of assembled contigs (N50) was revealed by majority of the contigs (>1,150 bp) (Table 2).

### 3.3. Gene Expression and Differential Expression Analysis

**Figure 3:**
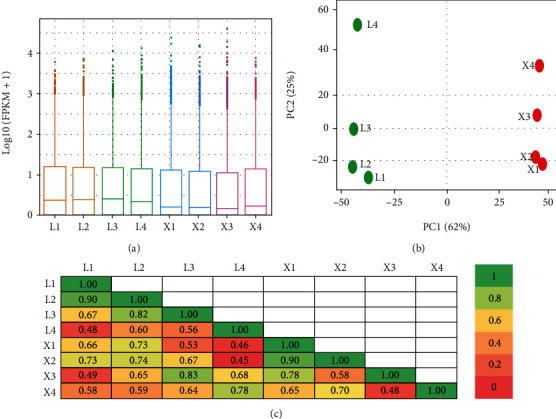
Relation among the biological samples of two *Nymphaea* species at four developmental stages revealed by expression evaluation with FPKM values (a), the principal component analysis (b), and correlation between samples (c). The four developmental stages are indicated by numeric 1 to 4 for *N. colorata*, as L1-L4, and *N. micrantha*, as X1-X4; the number in each box in (c) is the value of Pearson's coefficient of correlation, while color scale indicates its significance from 0 to 1.

The overall gene expression was higher in L-species than in viviparous X-species observed by FPKM values (Figure 3(a)). The principal component analysis (PCA) indicated the close relation of samples within species while a relative high distance between samples from the two species (Figure 3(b)). It was supported by the results of the average Pearson's coefficient of correlation (Figure 3(c)), which indicates extensive genetic dissimilarities between the two species at the different developmental stages.

**Figure 4:**
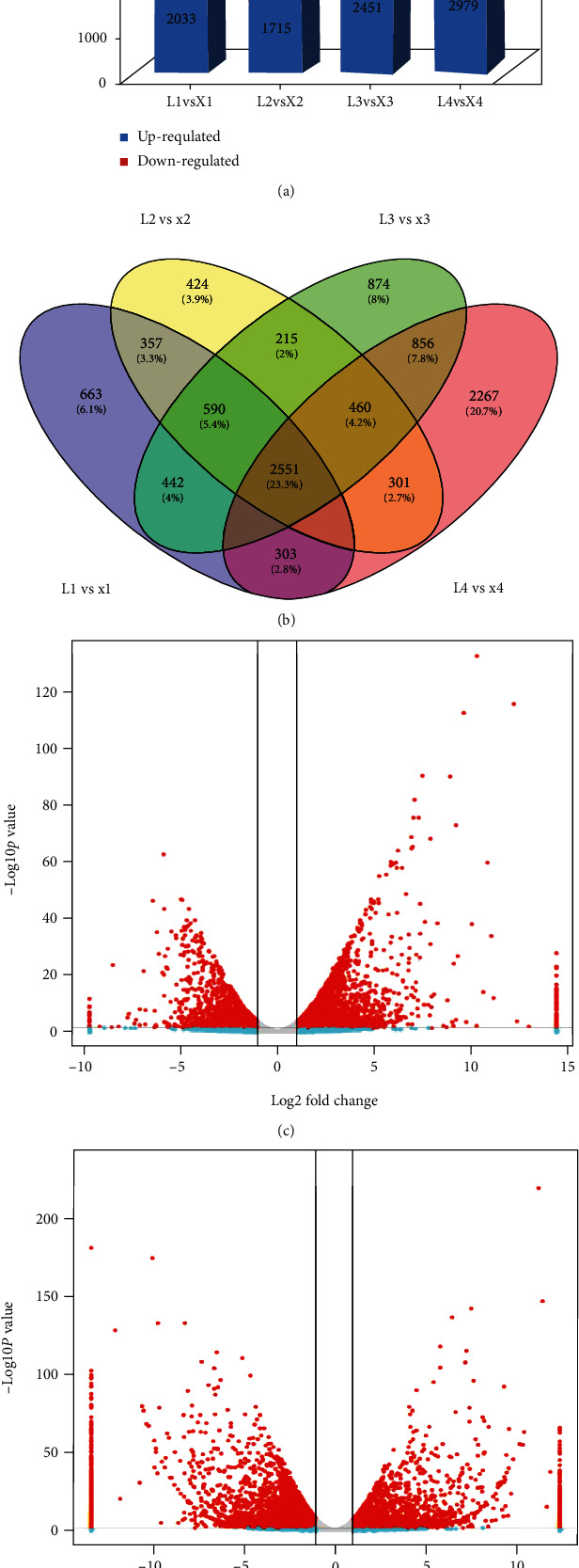
Summary of differentially expressed genes between two *Nymphaea* species. (a) The DEG between two *Nymphaea* species at four developmental stages, (b) the frequency of overlapping and unique DEG at four developmental stages, (c) the volcano graph of DEG in *N. micrantha* between its early and late developmental stages X1×X4, and (d) volcano graph for DEGs between two species at late developmental stage as L4×X4, where L represents the *N. colorata* and X represents *N. micrantha.*

The expressed genes were further screened for their differential expression (DEGs) using DESeq2 analysis based on |log 2 foldchange| ≥ 1 and false discovery rate (FDR) < 0.05. Relatively higher numbers of DEGs were regulated in later growth stages than the early stages (Figures 4(a) and 4(b)). The differential expression analysis between the two species showed 5,559, 5,164, 6,375, and 7,391 DEGs for L1 vs. X1, L2 vs. X2, L3 vs. X3, and L4 vs. X4, respectively, resulting in a total of 10,956 unique DEGs among the total expressed unigenes for all four stages (Figure 4(b); Additional Table S1). The majority of genes were downregulated with the relative growth in next developmental stages from X1 to X4 (Figure 4(c)). By comparing all of the DEGs from all developmental stages, we identified 2,551 (23.3%) core conserved genes constantly differentially expressed between the two species (Figure 4(d); Additional Table S1). Along with core conserved DEGs, a high number of specific DEGs (20.7%, 2,267) were observed between X- and L-species at the 4^th^ developmental stage, correlating with the appearance of viviparous leaves in X-species. These genes may represent key genes involved in viviparity trait in water lily.

### 3.4. Functional Annotation and Enrichment Analysis of DEGs

The differentially expressed genes were mapped to Gene Ontology (GO) terms in the GO database [25] for better understanding of functions and annotations in different developmental stages of viviparous and control *Nymphaea* species. GO functional enrichment analysis was performed adjusting *P* value of 0.05 as the cutoff (Additional Figure 1). A total of 4,943 GO terms were annotated to the 19,018 unigene hits (Additional Table S2). Among these terms, the maximum 52.26% (2,583) GO terms were belonged to the class “biological processes” (BP) followed by molecular functions (MF) (32.86%, 1,624 terms) and “cellular components” (CC) (11.77%, 582 terms). In CC, the most enriched GO terms were “nucleus” with 4,736 genes and membranous components including “integral components of membrane” with 3,909 genes and “plasma membrane” with 2,457 genes (Table 3), while in MF, most enriched terms were “ATP binding” with 3,123 genes followed by “metal ion binding” and “DNA binding transcription factor activity” with 2,793 and 1,062 genes, respectively. Among the biological processes, the “DNA integration” with 766 genes and “DNA recombination” with 735 genes were on top hit.

The DEGs were further evaluated for their functional enrichment between pairwise comparisons based on KEGG database [24]. The total 1,967 DEGs in the four developmental stages could be enriched in 173 unique KEGG pathways. At the early stages of plant development (L1 vs. X1 and L2 vs. X2), the DEGs related to “plant hormone signal transduction” and “flavonoid biosynthesis” pathways were downregulated, while the genes involved in “photosynthesis-antenna protein” and carbon fixation-related pathways were significantly upregulated among L- and X-species (Additional figure 2). The DEGs related to “base excision repair,” “DNA replication,” and various metabolism pathways were downregulated at the third developmental stage of both L- and X-species species, while the DEGs for photosynthesis, carbon fixation, and other biosynthesis pathways were still upregulated (Additional figure 2). During the fourth developmental stage, the DEGs for “fatty acid elongation,” “DNA replication,” “cell cycle,” and “meiosis” were differentially expressed (upregulated), while the genes for “flavonoid biosynthesis,” “fatty acid metabolism,” and various biosynthesis and metabolism-related pathways were also differentially expressed (downregulated) between the two species (Additional figure 1). Among all 173 annotated pathways, 127 pathways were conserved during all four developmental stages of X- and L-species (Table 4 and Additional Table S3).

### 3.5. Plant Hormone Signal Transduction

**Figure 5:**
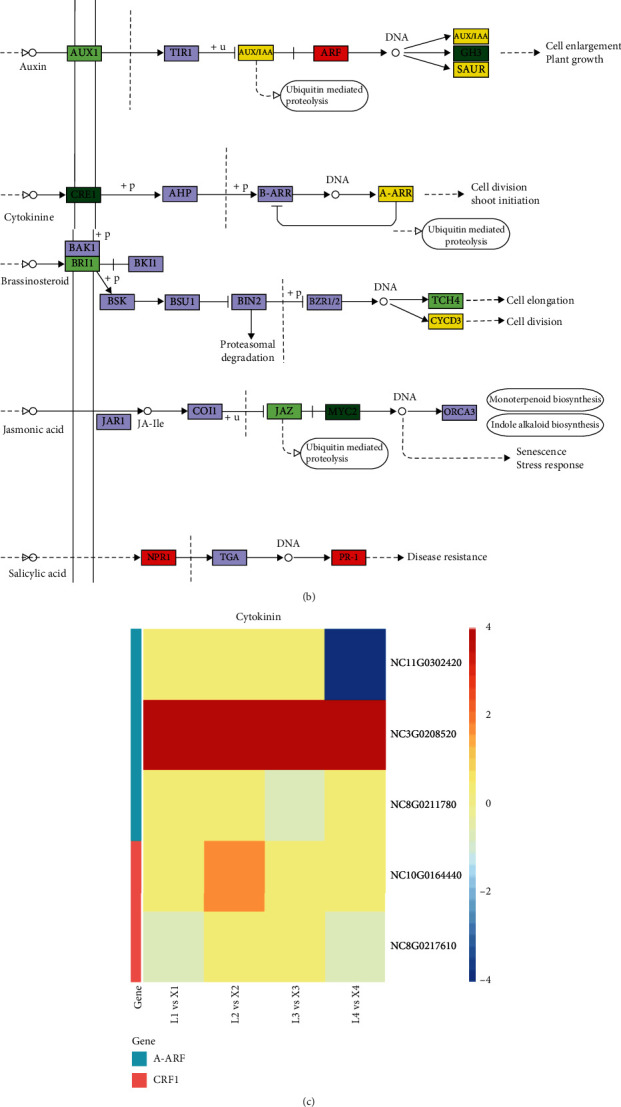
Regulation of plant hormone signaling pathway in viviparous and nonviviparous *Nymphaea* species. Heatmap based log 2 FC values of DEGs related to auxin (a), cytokinin (c), and brassinosteroid, jasmonic acid and salicylic acid (d) in all four developmental stages. Pathway map (b) showing the differential regulation of plant hormone signaling pathway between *N. micrantha* and *N. colorata*. In pathway map (b), the genes highlighted in green are downregulated, in red are upregulated, and in yellow are up-/downregulated DEGs.

**Figure 6:**
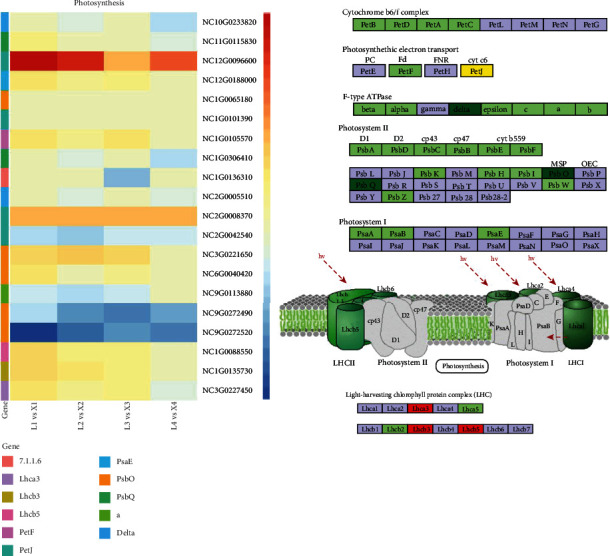
Regulation of photosynthesis pathway in viviparous and nonviviparous *Nymphaea* species. Heatmaps showing log 2 FC values of DEGs in all four developmental stages. Pathway map showing the differential regulation of photosynthesis related pathways between *N. micrantha* and *N. colorata*. The genes highlighted in green, red, and yellow colors represent down-, up -, and up-/downregulated DEGs.

Among the highly conserved pathways in all four developmental stages, the plant hormone signal transduction pathway showed the highest differential regulation. Similar results have been reported in response to biotic stress in other plants [30]. The auxin-induced proteins (AUX/IAA) were differentially regulated in X- and L-species at various stages. The auxin response factor (ARF) transcriptional factor was significantly upregulated which resulted in suppression of small auxin-up RNA (SAUR) gene family in L-species. The *glycoside hydrolase 3* (*GH3)* expression was continuously decreased in L-species at later growth stages, indicating that L-species was unable to continue cell enlargement and plant growth (Figure 5). Along with auxins, the cytokinin signaling-related gene *histidine kinase* (*CRE1*) was downregulated, and type A ARR was differentially expressed for signaling regulation. The downregulation of these genes in L-species is indicative of lower cytokinin signaling in L-species for cell division and shoot initiation in later developmental stages (Figure 6). We further identified the differential regulation of cyclin-D3 (CYCD3) protein which is responsible for cell division and mitotic cycles in leaves [31]. The gene for MYC2 family was also downregulated in L-species which are well known to shape the plant growth and development [32]. Together with the higher auxin, cytokinin, and jasmonic acid contents in the leaves of X-species, the upregulation of key DEGs benefits the development of leaf outgrowth at later plant developmental stages. The genes related to Natriuretic Peptide Receptor 1 (NPR1) and pathogenesis-related protein (PR1) were upregulated in L-species which may be involved in the adenosine triphosphate (ATP) synthesis and responses to various stresses [33, 34] (Figure 5).

### 3.6. Carbon Fixation and Photosynthesis

The plant development is also affected by the energy-related pathways including the changes in photosynthetic efficiency in response to modifications in photosynthesis-antenna proteins, carbon fixation pathways, and the photosynthesis pathway [35]. The modifications in photosynthesis and carbon fixation-related pathways are accompanied by other energy and metabolism-related pathways including galactose metabolism, starch and sucrose metabolism, nitrogen metabolism, pentose phosphate pathway, citrate cycle, and carbon fixation in photosynthetic organisms [36]. The DEGs between L- and X-species were enriched in these pathways signifying large-scale transcriptional changes in energy-related pathways. Three DEGs in light harvesting chlorophyll protein complex (LHC) were observed to be significantly upregulated. Seven photosystem II (PSII) proteins (PsbK, PsbH, PsbI, PsbW, PsbZ, PsbO, and PbsQ) and one photosystem I (PSI) protein (PsaE) were differentially regulated. Only two genes related to “delta” and “a” F-type ATPase were downregulated. Four genes in photosynthesis electron chain transport (PetJ and PetF) were differentially expressed in the two species (Figure 6).

**Figure 7:**
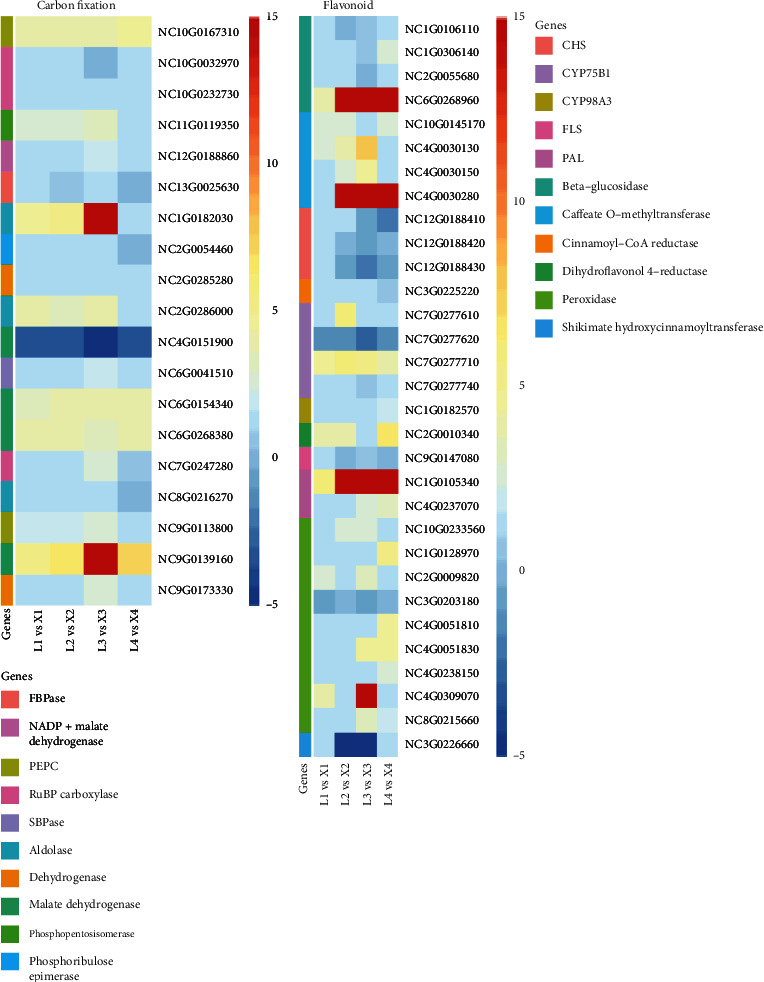
Heatmaps of log2 fold change values of differentially expressed genes involved in carbon fixation pathway and the flavonoid biosynthesis pathways commonly observed in all four developmental stages of *Nymphaea* species.

Twenty DEGs (12 upregulated, 7 downregulated, and one up-/downregulated in at least one growth stage of development in the two species) were enriched in carbon fixation pathway in photosynthetic organisms (Figure 6). Two *dehydrogenase*, four *malate dehydrogenase* (*MDH*), one *NADP+ malate dehydrogenase* genes were highly downregulated in L-species; however, other *MDH*s were upregulated. One *FBPase* and one *SBPas*e genes were downregulated. Two aldose-related genes were downregulated at the 4^th^ developmental stage, while one *phosphoenolpyruvate carboxylase (PEPC*) gene was upregulated and one downregulated at the 4^th^ stage of plant development. Meanwhile, two *phosphoribulose epimerase carboxylase* (*RuBPC*) genes were downregulated, and one gene was upregulated throughout the plant development. The differential regulation patterns of various genes with the same annotation within the energy-related pathways showed complex differential transcription signatures in *Nymphaea* species. These expression changes in these pathways indicate their importance in the viviparous growth at later plant growth stages (Figure 7).

### 3.7. Phenylpropanoid/Flavonoid Biosynthesis

Phenylpropanoids and flavonoids play vital roles in plant development by acting as essential components of cell walls, protectants against high light and UV radiation, phytoalexins against herbivores and pathogens, and floral pigments to mediate plant-pollinator interactions [37]. Nineteen DEGs were upregulated, eleven downregulated, and one up-/downregulated in the two *Nymphaea* species. Among the enriched proteins in these pathways, four *beta-glucosidase*, three *chalcone synthase* (*CHS*), one *flavonol synthase* (*FLS*), and one *shikimate-hydroxycinnamoyl-transferase* genes were downregulated. Two *phenylalanine ammonia lyase* (*PAL*), eight *peroxidase*, and four *caffeate-O-methyltransferase* genes were upregulated (Figure 7). These complex mechanisms of transcriptome expression reveal the importance of these DEGs in the differential development of viviparity in X-species than in L-species.

### 3.8. Validation of RNA Analysis by qRT-PCR

**Figure 8:**
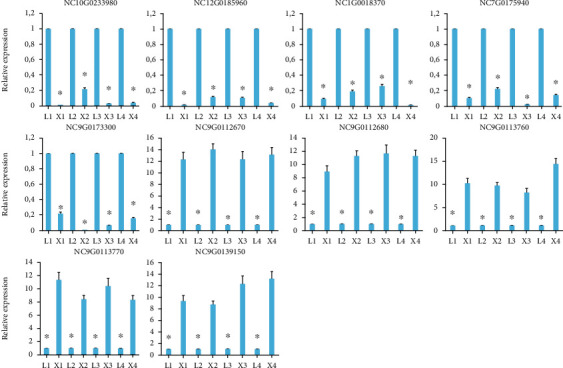
qRT-PCR validation of ten selected differentially expressed genes. The error bar represents standard deviation (SD) of three technical and biological replicates. ∗ means the gene expression between the two species at each growth stage was significantly (*P* ≤ 0.05) different. The four developmental stages are indicated by numeric 1 to 4 for *N. colorata* (L) as L1-L4 and *N. micrantha* (X) as X1-X4.

To further validate the expression of the identified DEGs between the two *Nymphaea* species at different developmental stages, we selected top ten DEGs (top five positively and top five negatively expressed genes) and performed qRT-PCR expression profiling. The qRT-PCR results of the selected genes were almost consistent with that of RNA-seq analysis (Figure 8). There was significant difference (*P* ≤ 0.05) of expression levels between the two studied species for all evaluated genes at the four growth stages. This result supports the DEG analysis and subsequent interpretations.

## 4. Discussion

### 4.1. Transcriptome Enrichment and Genetic Basis of Viviparity in Water Lily

The extrachromosomal genome [38], transcriptome, and proteome [10] analyses for various morphological and physiological traits have been reported in water lilies. However, this is the first research focused on the molecular basis of viviparous growth in water lilies. We generated the whole transcriptome sequence from leaf tissues at various plant developmental stages, assembled the sequencing data and annotated the differentially expressed for functional assessment. The vivipary reproduction has a great flexibility for parental control of embryonic development, which in turn allows viviparous organisms to reproduce successfully in adverse environments [39]. There are some studies reported for the genetic bases of transition from oviparity to viviparity in animals by comparing genomic and transcriptomic data [39], but our knowledge of viviparity trait in plants is still limited. We used the *N. micrantha* as a model viviparous water lily to compare its transcriptome with the nonviviparous *N. colorata* at various developmental stages. The highest number of DEGs between the two species was observed at the 4^th^ developmental stage of water lily. The viviparity phenomenon was observed to be associated with 127 highly conserved metabolic pathways in the four developmental stages. The top enriched pathways belong to plant hormone signal transduction, DNA replication, cell cycle, photosynthesis, and carbon fixation. The expression analyses of differentially expressed genes (DEG) indicate the complex network underlying the viviparous growth [40]. Besides, there were 3,574 DEGs conserved between the third and fourth growth stages that may be involved in plantlet development and may provide clues for further studies on identifying inducible/specific mechanisms/components involved in adaptation of viviparous growth.

### 4.2. Effect of Phytohormones on Viviparous Growth in Water Lily

As plants grow, they develop new organs as primary and secondary leaves, lateral roots, and flowers [41]. The plant development is regulated by complex hormone interaction and signaling which helps various species to evolve in the wide range of environmental conditions [41]. Various biotic/abiotic stress combinations develop a new type of signal and response in plants, resulting in a novel transcription signature [42]. Through our comparative transcriptome study among the viviparous and nonviviparous water lily species, we found similar results as the biotic stress responses [43]. The biotic/abiotic stress can cause the variation in phytohormonal balance in plants. We identified the significant differential regulation of *AUX*/*IAA*, *ARF*, *GH3*, and *SAUR*. *Auxin/Indole-3-Acetic acid* (*Aux/IAA*) genes are the early response genes that trigger gene reprogramming precisely and rapidly under stress [44]. Auxin response factor (ARF) transcription factors are activated upon auxin perception and initiate downstream signaling pathways including the small auxin upregulated RNA (*SAUR*) genes [41]. *SAUR*s regulate many auxin-mediated responses, specifically the tissue growth via cell elongation [41]. We identified the highly conserved genes for ARF (*NC0286500*) and *SAUR*s (*NC14G0174900, NC263970, NC7G0235790,* and *NC9G0168130*) (Figure 5). These genes may directly be involved in the development of viviparous outgrowth in water lilies by their involvement in cell division, enlargement, and differentiation [44]. The auxin in association with cytokinins also helps in cell differentiation [41]. The higher expression of CRE cytokinin may result in viviparous cell differentiations as CRE is known for acting on shoot apical meristem differentiation [45]. The changes in other signaling hormones also have their indirect role to enhance the viviparous plantlet growth. It is possible because CYCD3 cyclins was previously reported for mitotic cell divisions [31]. The upregulated expression of DEGs related to jasmonic acid (JA) signaling, i.e., *MYC2*, is also an indicator of high response to light phytochromes [46] that was reduced in L-species. It can finally be concluded that the viviparous plantlet differentiation could be due to the higher regulation of auxin and cytokinin and subsequent activation of other relevant phytohormones (Figure 5). In previous studies, the role of abscisic acid (ABA) and gibberellic acid (GA) has also been reported for development of viviparity and influence on meristematic tissue, but in the present study, no DEG was observed to be involved in ABA or GA-based function for viviparity.

### 4.3. Involvement of Viviparous Plantlet in Photosynthesis

Development of viviparous plantlet growth significantly affects the photosynthesis and subsequent energy-related processes [47]. The increasing expression of light harvesting chlorophyll protein complex (*LHC3*) and *LHC5* could be a subsequent regulation effect of viviparity. Further, the downregulation of relatively higher number of photosynthesis associated proteins (photosystem II (PSII) and photosystem I (PSI) proteins) in L-species reduced its photosynthetic potential that possibly affected downstream energy metabolism-related pathways [48, 49]. The *PsbQ* and *PsbO* proteins of PSII-complex were downregulated in L-species, while they have higher expression in X-species (Figure 6). These proteins have been reported for their role to stabilize the interaction between the membrane-bounded PSII subunits and the related proteins, i.e., *PsbP*. Hence, they may be involved in the regulation and evolution of photosynthesis in viviparous plantlet.

### 4.4. Role of Flavonoid Pathways in Viviparous Water Lily

Phenylpropane and peroxidases are the precursor of lignin biosynthesis, which are an essential element of cell wall [50]. The phenylpropanoids are a group of plant secondary metabolites derived from phenylalanine and have a wide variety of functions both as structural and signaling molecules [51]. Lignin is derived from phenylalanine and has a wide variety of functions both as structural and signaling molecules [51, 52]. The DEGs for peroxidases, caffeate O-methyltransferase, CYP75B1, and PAL were upregulated in the nonviviparous species (Figure 7) of water lily. These secondary metabolites are known to play key roles in inhibition of seed germination and reduced water permeability [50]. Inversely, the structural genes (CHS) [53] and genes related to beta glucosidase and FLS which are known for tissue development and the anthocyanin biosynthesis [54, 55] showed downregulation in nonviviparous species (Figure 7). The higher expression of these genes in viviparous species revealed their importance in the viviparity phenomenon.

## 5. Conclusions

The comparison of transcriptome of viviparous species *N. micrantha* and nonviviparous species *N. colorata* of water lilies revealed the variation in expression of various genes indicating their putative role in viviparity. *Nymphaea micrantha* showed upregulated genes for plant hormone signal transduction including *AUX/IAA*, *ARF*, *GH3*, and *SAUR* gene families. Regulation of these genes involved in cell division, elongation, and differentiation showed their association to viviparity in *N. micrantha*. The increased expression levels of these genes in *N. micrantha* triggered downstream phytohormone signaling cascade as noticed by the regulation of genes such as *MYC2*s and *CYCD3*. The viviparity in plants also modulates changes in the important energy-related pathways. Genes related to both light harvesting chlorophyll complex and photosystem I and photosystem II were differentially expressed in both species. Importantly, increased expression of major genes related to the above cited pathways in *N. micrantha* possibly regulates the phenomenon of viviparity. Overall, the transcriptomic signatures identified in this study are important basis for future research of viviparity in water lilies and other plant species.

## Figures and Tables

**Table 1:** Data summary for de novo transcriptome assembly of *Nymphaea* species.

	Raw		Cleaned		Q30 (%)	GC (%)
	Reads	Bases	Reads	Bases		
*N. micrantha*						
X1	54,220,545	8,133,081,750	51,993,151	7,798,972,650	95.01	49.50%
X2	58,697,453	8,804,617,950	56,564,881	8,484,732,150	94.98	49.50%
X3	54,706,731	8,206,009,650	52,022,433	7,803,364,950	94.58	50.00%
X4	56,806,450	8,520,967,500	54,658,273	8,198,740,950	94.34	49.00%
*N. colorata*						
L1	61,757,662	9,263,649,300	59,122,075	8,868,311,250	94.81	49.00%
L2	58,723,860	8,808,579,000	56,770,472	8,515,570,800	94.95	50.00%
L3	51,641,067	7,746,160,050	49,703,777	7,455,566,550	94.78	50.00%
L4	53,359,196	8,003,879,400	51,107,727	7,666,159,050	94.79	50.00%

**Table 2:** Characteristic descriptive of de novo transcriptome assembly of *Nymphaea* species.

Descriptive	Value
Total length (bp)	99,484,039
Total number	114,762
N50 (bp)	1150
Average (bp)	866.87
Minimum (bp)	301
Maximum (bp)	15,206
Number of contigs ≥ 300 bp	114,762
Number of contigs ≥ 500 bp	68,559
Number of contigs ≥ 1000 bp	31,663

**Table 3:** Gene Ontology (GO) classification of unique top twenty enriched GO terms among four developmental stages of *Nymphaea* species.

GO ID	GO term	GO class	DEGs
GO:0005634	Nucleus	cellular_component	4736
GO:0016021	Integral component of membrane	cellular_component	3909
GO:0005524	ATP binding	molecular_function	3123
GO:0046872	Metal ion binding	molecular_function	2793
GO:0005829	Cytosol	cellular_component	2682
GO:0005737	Cytoplasm	cellular_component	2463
GO:0005886	Plasma membrane	cellular_component	2457
GO:0009507	Chloroplast	cellular_component	1886
GO:0003677	DNA binding	molecular_function	1366
GO:0005739	Mitochondrion	cellular_component	1152
GO:0003700	DNA-binding transcription factor activity	molecular_function	1062
GO:0004190	Aspartic-type endopeptidase activity	molecular_function	1001
GO:0005576	Extracellular region	cellular_component	877
GO:0005794	Golgi apparatus	cellular_component	816
GO:0003676	Nucleic acid binding	molecular_function	795
GO:0003723	RNA binding	molecular_function	784
GO:0003964	RNA-directed DNA polymerase activity	molecular_function	768
GO:0015074	DNA integration	biological_process	766
GO:0005789	Endoplasmic reticulum membrane	cellular_component	742

**Table 4:** List of pathways highly enriched with differentially expressed genes between X1 and X4 growth stages of *N. micrantha.*

KEGG ID	Pathway	*P* value	DEGs
ko00591	Linoleic acid metabolism	0	3
ko00940	Phenylpropanoid biosynthesis	0.00000021	18
ko00941	Flavonoid biosynthesis	0.00000109	10
ko00196	Photosynthesis-antenna proteins	0.0000184	4
ko00565	Ether lipid metabolism	0.0000184	4
ko00944	Flavone and flavonol biosynthesis	0.0000184	4
ko00908	Zeatin biosynthesis	0.00016421	3
ko00062	Fatty acid elongation	0.00024383	7
ko00073	Cutin, suberine, and wax biosynthesis	0.00038521	6
ko00270	Cysteine and methionine metabolism	0.00047493	12
ko04075	Plant hormone signal transduction	0.00060961	16
ko00830	Retinol metabolism	0.00074745	3
ko00982	Drug metabolism-cytochrome P450	0.00093032	7
ko04540	Gap junction	0.00161368	6
ko00350	Tyrosine metabolism	0.00278399	5
ko01040	Biosynthesis of unsaturated fatty acids	0.00278399	5
ko00592	Alpha-linolenic acid metabolism	0.00609769	5
ko00980	Metabolism of xenobiotics by cytochrome P450	0.00609769	5
ko00140	Steroid hormone biosynthesis	0.00791195	3
ko00564	Glycerophospholipid metabolism	0.01020997	7

## Data Availability

The RNA-seq raw data has been submitted to NCBI GEO under the accession number GSE164888.

## Supplementary Materials

Supplementary Materials Additional Figure 1: list of top enriched Gene Ontology (GO) terms between two *Nymphaea* species *N. micrantha* and *N. colorata*, at four developmental stages. Additional Figure 2: list of top twenty enriched KEGG pathways between two *Nymphaea* species *N*. *micrantha* and *N. colorata*, at four developmental stages. Additional Table S1: list of unique differentially expressed genes among four developmental stages of *N. micrantha* (X) and *N. colorata* (L) species. Available at https://figshare.com/articles/dataset/Additional_information_tables_xlsx/19688034). Additional Table S2: list of unique Gene Ontology (GO) terms annotated to the differentially expressed genes among four developmental stages of *N. micrantha* (X) and *N. colorata* (L) species. Available at https://figshare.com/articles/dataset/Additional_information_tables_xlsx/19688034. Additional Table S3: list of highly conserved KEGG pathways annotated to the differentially expressed genes among four developmental stages of *N. micrantha* (X) and *N. colorata* (L) species. Available at https://figshare.com/articles/dataset/Additional_information_tables_xlsx/19688034. Additional Table S4: primer sequences of the genes used in the qRT-PCR. Available at https://figshare.com/articles/dataset/Additional_information_tables_xlsx/19688034.
