# Supplementary figures and images for: Transcriptomic Insight into Viviparous Growth in Water Lily
Source: Biomed Res Int. 2022 Jul 7;2022:8445484. doi: 10.1155/2022/8445484 (PMC9283058; doi:10.1155/2022/8445484)

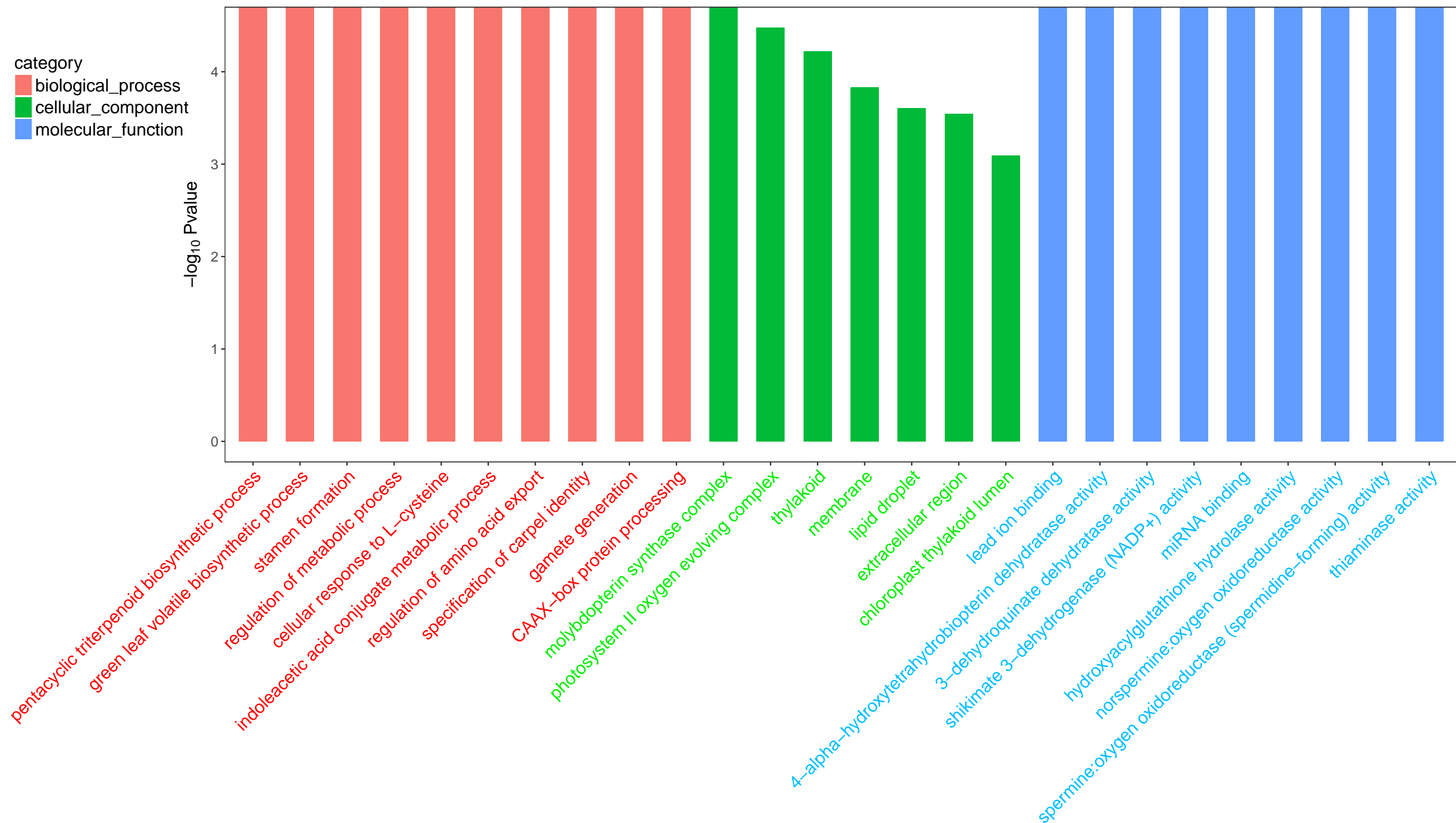

Supplement: Supplementary Materials — Additional Figure 1: list of top enriched Gene Ontology (GO) terms between two Nymphaea species N. micrantha and N. colorata, at four developmental stages. Additional Figure 2: list of top twenty enriched KEGG pathways between two Nymphaea species N. micrantha and N. colorata, at four developmental stages. Additional Table S1: list of unique differentially expressed genes among four developmental stages of N. micrantha (X) and N. colorata (L) species. Available at https://figshare.com/articles/dataset/Additional_information_tables_xlsx/19688034). Additional Table S2: list of unique Gene Ontology (GO) terms annotated to the differentially expressed genes among four developmental stages of N. micrantha (X) and N. colorata (L) species. Available at https://figshare.com/articles/dataset/Additional_information_tables_xlsx/19688034. Additional Table S3: list of highly conserved KEGG pathways annotated to the differentially expressed genes among four developmental stages of N. micrantha (X) and N. colorata (L) species. Available at https://figshare.com/articles/dataset/Additional_information_tables_xlsx/19688034. Additional Table S4: primer sequences of the genes used in the qRT-PCR. Available at https://figshare.com/articles/dataset/Additional_information_tables_xlsx/19688034. [file 8445484.f1.zip › Additional Figure 1.pdf]

# KEGG Enrichment top 20

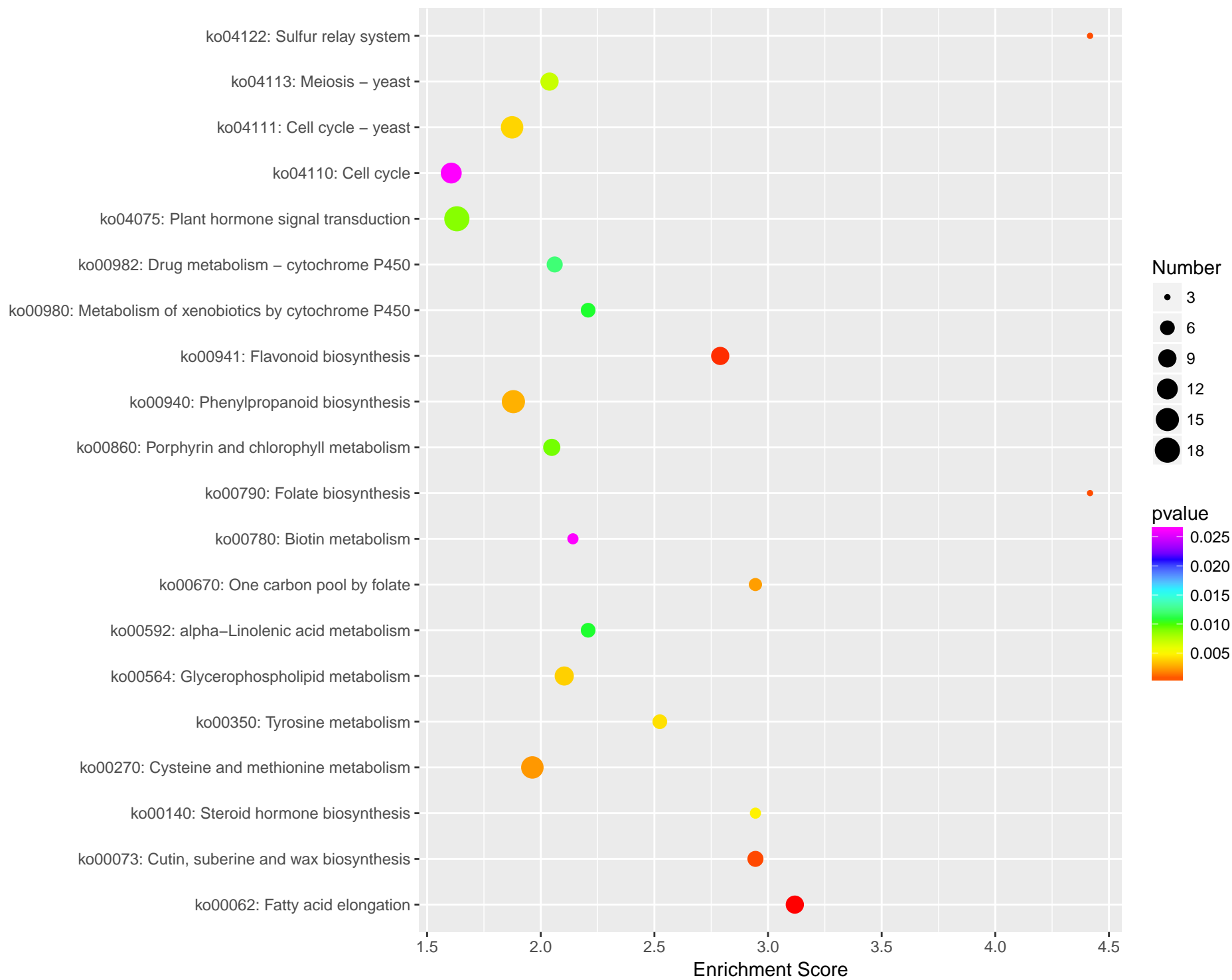

Supplement: Supplementary Materials — Additional Figure 1: list of top enriched Gene Ontology (GO) terms between two Nymphaea species N. micrantha and N. colorata, at four developmental stages. Additional Figure 2: list of top twenty enriched KEGG pathways between two Nymphaea species N. micrantha and N. colorata, at four developmental stages. Additional Table S1: list of unique differentially expressed genes among four developmental stages of N. micrantha (X) and N. colorata (L) species. Available at https://figshare.com/articles/dataset/Additional_information_tables_xlsx/19688034). Additional Table S2: list of unique Gene Ontology (GO) terms annotated to the differentially expressed genes among four developmental stages of N. micrantha (X) and N. colorata (L) species. Available at https://figshare.com/articles/dataset/Additional_information_tables_xlsx/19688034. Additional Table S3: list of highly conserved KEGG pathways annotated to the differentially expressed genes among four developmental stages of N. micrantha (X) and N. colorata (L) species. Available at https://figshare.com/articles/dataset/Additional_information_tables_xlsx/19688034. Additional Table S4: primer sequences of the genes used in the qRT-PCR. Available at https://figshare.com/articles/dataset/Additional_information_tables_xlsx/19688034. [file 8445484.f1.zip › Additional Figure 2.pdf]
